# Ghassoul-Clay- and Oil-Loading-Induced Colloidal Gelation of Lavender Essential Oil Emulsified Systems

**DOI:** 10.3390/gels12080689

**Published:** 2026-08-03

**Authors:** Barbara Bigi, Stefania Petralito, María Jesús Rodriguez-Palero, Luis Alfonso Trujillo-Cayado, Jenifer Santos

**Affiliations:** 1 Department of Chemistry and Technologies of Drug, “Sapienza” University of Rome, Piazzale Aldo Moro, 5, 00185 Rome, Italy; barbara.bigi@uniroma1.it (B.B.); stefania.petralito@uniroma1.it (S.P.); 2 Departamento de Ciencias de la Salud y Biomedicina, Facultad de Ciencias de la Salud, Universidad Loyola Andalucía, Avda. de las Universidades s/n, Dos Hermanas, E41704 Sevilla, Spain; mjrodriguez@uloyola.es; 3 Departamento de Ingeniería Química, Escuela Politécnica Superior, Universidad de Sevilla, c/Virgen de África, 6, E41007 Sevilla, Spain

**Keywords:** colloidal gel, emulsion gel, Ghassoul clay, lavender essential oil, viscoelasticity, weak gel, shear-thinning, droplet crowding, physical stability, multiple light scattering

## Abstract

Lavender essential oil is a promising bioactive ingredient, but its incorporation into aqueous formulations is limited by its low water solubility and high volatility. This work structured lavender oil-in-water emulsified systems through two approaches: Ghassoul clay incorporation and increased oil loading. The standard formulation containing 1.25 wt.% oil showed a narrow droplet size distribution, with a Sauter mean diameter of 114 nm, respectively, and Newtonian behavior with a viscosity of 0.97 mPa·s. Ghassoul addition did not significantly modify droplet size, but induced shear-thinning and gel-like behavior. Zero-shear viscosity increased from 2 Pa·s at 5 wt.% clay to 550 Pa·s at 20 wt.%, while 15–20 wt.% Ghassoul showed the highest resistance to clay sedimentation after 30 days. Increasing lavender oil concentration promoted droplet growth and broader size distributions. The 30 wt.% oil formulation exhibited the strongest droplet-mediated structure, with G′ exceeding G″ and zero-shear viscosity reaching 156 Pa·s, whereas 40 wt.% oil weakened the network due to droplet coarsening and polydispersity. The results highlight the key role of clay-particle networking, droplet crowding, droplet-size distribution, and viscoelastic structuring in controlling gel-like behavior and physical stability, supporting the design of essential oil-based colloidal gels for topical, pharmaceutical, or cosmetic delivery applications.

## 1. Introduction

Nanoemulsions represent an extensively employed vehicle for hydrophobic active compounds, such as essential oils (EOs), able to enhance their stability and allow their use in aqueous environments. EOs have gained relevance due to their various properties of interest, such as anti-inflammatory and antioxidant activity [1,2]. In particular, they possess antimicrobial attributes considered valuable in the context of antimicrobial resistance phenomena (AMR) since their natural origin [3]. The low water solubility and high volatility of essential oils make nanoemulsification particularly advantageous for enhancing their effectiveness as biocidal agents across a range of applications [4,5].

In recent years, oil-in-water nanoemulsions have garnered growing scientific interest as effective biocidal platforms for delivering essential oils, such as eugenol, cinnamaldehyde, thymol, and carvacrol, which exhibit broad-spectrum antimicrobial properties [6]. Oil-in-water (O/W) nanoemulsions, in particular, are widely used for encapsulating essential oils due to their nanometric droplet size, which increases the specific surface area, improves dispersion in aqueous environments, and protects volatile compounds, thereby enhancing their stability and bioavailability [7,8]. Such systems typically include an oil phase, surfactant or surfactant blends, and an aqueous phase, with the surfactant-to-oil ratio (SOR) playing a pivotal role in achieving stable formulations and controlling droplet size and physicochemical behavior [9]. In these formulations, nanometric oil droplets stabilized by surfactants enhance not only the dispersibility and chemical stability of the oils, but also their interaction with microbial membranes, thereby boosting their antimicrobial efficacy [10,11].

Nonetheless, the fabrication of stable nanoemulsions demands close formulation control due to potential instability mechanisms such as Ostwald ripening, which can lead to droplet growth and coalescence over time [12,13]. These issues may be mitigated by using appropriate lipid surfactant, such as soybean-derived phosphatidylcholine (SoyPC), in mixtures with non-lipid ones, that provide both electrostatic and steric stabilization, as well as by modifying the dispersing aqueous phase to increase the overall viscosity of the system, thereby hindering droplet mobility and collision [14,15,16]. SoyPC stands out as a particularly advantageous emulsifier, offering a unique combination of amphiphilic properties and physiological compatibility. As a naturally occurring phospholipid, phosphatidylcholine integrates seamlessly into biological membranes, enhancing its biocompatibility and facilitating the delivery of encapsulated actives through improved membrane interaction and permeability [17]. In high-energy emulsification methods, such as high-pressure homogenization or ultrasonication, soy phosphatidylcholine contributes significantly to droplet size reduction and colloidal stability due to its ability to rapidly adsorb at the oil–water interface and form a robust interfacial film. This film effectively prevents droplet coalescence and Ostwald ripening, promoting long-term kinetic stability [18,19]. Furthermore, the use of soy phosphatidylcholine aligns with clean-label and natural formulation trends, as it is non-toxic, biodegradable, and derived from a renewable plant source. This makes it particularly suitable for pharmaceutical, cosmetic, and nutraceutical applications where safety and natural origin are paramount [17].

In this regard, it is not sufficient to optimize encapsulation and droplet size alone. The flow properties of the nanoemulsion, particularly viscosity, play a crucial role in determining the practical performance of these systems in real-world applications [20]. In many cases, especially for topical, mucosal, or surface disinfection uses, there is a clear need to increase formulation viscosity. Higher viscosity nanoemulsions show superior retention on vertical or irregular surfaces such as wounds, surgical tools, mucosal linings, or catheters, which enhances the contact time between the active compound and microbial contaminants, thereby increasing biocidal efficacy [21]. Additionally, higher viscosity minimizes the risk of uncontrolled run-off and spread, supporting localised and safer application, especially in sensitive clinical settings. Viscous formulations also allow for sustained release of active agents, extending their antimicrobial effect over time, which is particularly advantageous for wound dressings, implant coatings, and mucosal antiseptics [21,22,23]. The enhanced rheological properties also translate into a better user experience, including easier handling, reduced splashing, and more precise dosing, which are important for products such as antiseptic gels, oral formulations, or catheter site disinfectants [21].

To achieve these rheological goals, several variables can be modulated, with the percentage of oil being one of the most influential. Increasing oil concentration from low (e.g., 5 wt.%) to high values (e.g., 40 wt.%) significantly raises the viscosity of the nanoemulsion, due to increased droplet crowding and inter-droplet interactions, and may lead to non-Newtonian behaviors such as shear-thinning flow [24]. These changes necessitate corresponding adjustments in surfactant concentration to maintain droplet stability and homogeneity [25]. Thus, another viable strategy for controlling viscosity involves modifying the type or amount of surfactant, or incorporating viscosity-enhancing excipients, such as polymers, hydrocolloids or even clays, into the aqueous phase [26,27,28]. The presence of co-surfactants and structured interfacial layers, including lamellar or liquid crystalline phases, contributes to increased viscosity and improved resistance to coalescence [29].

In this context, the incorporation of clays as rheological modifiers in emulsified systems has gained increasing interest due to their natural origin, unique colloidal behavior and surface properties. Ghassoul clay, also known as Rhassoul, is a naturally occurring mineral clay belonging to the smectite group and is primarily composed of hydrated magnesium aluminum silicates. Its chemical composition typically includes silicon, aluminum, magnesium, iron, and calcium, and it exhibits a layered phyllosilicate structure [30]. In aqueous environments, Ghassoul undergoes swelling through the intercalation of water molecules between its silicate layers, leading to the delamination of stacked lamellae into individual platelets or tactoid structures [31]. This results in the formation of stable colloidal suspensions containing anisotropic, plate-like particles with a high aspect ratio and a net negative surface charge, particularly on the basal planes. These dispersed lamellae can organize into three-dimensional colloidal networks driven by electrostatic repulsion and entropic forces, imparting thixotropic and pseudoplastic properties to the system [32]. The extent of swelling, exfoliation, and structural organization depends on factors such as pH, ionic strength, and clay concentration. Although Ghassoul clay is not a classical hydrocolloid like xanthan or guar gum, it can function as an effective rheological agent by increasing the viscosity of the continuous phase through the formation of these colloidal networks.

In emulsified systems, particularly oil-in-water nanoemulsions, Ghassoul can also contribute to interfacial stabilization. In the presence of oppositely charged surfactants, the negatively charged clay platelets may adsorb onto the positively charged oil droplets via electrostatic attraction. This interaction can create a steric barrier around the droplets, reducing coalescence and enhancing stability [32,33]. The mechanism bears similarities to Pickering emulsions, where solid particles stabilize emulsions by forming a physical shell at the interface. However, in the case of clay-modified emulsions, stabilization arises from a combination of steric hindrance and electrostatic effects, which are highly dependent on the surface charge of the droplets, the type and concentration of surfactants, and the amount of clay added. Improper charge matching or excessive clay can result in flocculation through bridging or charge neutralization, ultimately compromising colloidal stability [30,31,33,34]. Therefore, while Ghassoul clay offers valuable functional properties in both rheological modification and interfacial stabilization, its successful use in formulations requires precise control of composition and compatibility among the system’s components.

Numerous studies have demonstrated the significant influence of formulation parameters on the performance of essential oil–based nanoformulations. For instance, in cinnamon leaf oil nanocreams, higher oil content and the presence of fatty alcohols such as cetostearyl alcohol were shown to increase yield stress, promote plastic flow behavior, and induce thixotropy—thereby improving skin application and adhesion [35]. Likewise, nanoemulsions of oregano essential oils have demonstrated Newtonian or shear-thinning rheological profiles depending on surfactant concentration and interfacial structuring [36]. While low-viscosity nanoemulsions are beneficial for spraying or irrigation applications, they often present limited adhesion and retention on mucosal or skin surfaces [37]. In contrast, gelling agents substantially enhance viscosity, which slows the release of volatile constituents such as eugenol or cinnamaldehyde and extends antimicrobial efficacy, from a few hours to up to 24 h in cinnamon oil nanoemulgel systems for oral use [37]. In these systems, the increased viscosity supports prolonged retention at mucosal sites and enables controlled, sustained diffusion of active compounds. Importantly, even low-viscosity formulations, when optimized for droplet size and surfactant composition, can achieve significantly higher antimicrobial activity than bulk essential oils, often showing enhanced microbial penetration and stability [37].

From a gel-science perspective, concentrated emulsions and particle-containing emulsified systems may be regarded as colloidal gels when droplet–droplet interactions, particle networking, or mixed droplet–particle associations generate a continuous stress-bearing structure. In these systems, the transition from a liquid-like nanoemulsion to a gel-like material is governed not only by droplet size and dispersed-phase volume fraction, but also by the formation of percolated colloidal networks able to store elastic energy, resist flow at low deformation, and restrict gravitational destabilization. Therefore, evaluating the relationship between formulation composition, viscoelastic response, zero-shear viscosity, and physical stability is essential for understanding the gelation mechanisms of essential oil-based emulsified systems.

In this work, we aim to formulate *Lavandula angustifolia* essential oil into oil-in-water emulsified systems with different oil concentrations or in the presence of a precise amount of Ghassoul clay. We employed SoyPC as a lipid surfactant in mixtures with no lipid surfactant, benzalkonium chloride, to prepare nanoemulsions by a high-shear mixer and ultrasonication procedure (HSM-US). The rational design of oil-in-water nanoemulsions for biocidal applications must integrate both physicochemical and functional considerations. By systematically adjusting oil content, surfactant type and concentration, and the use of aqueous-phase modifiers, it is possible to fine-tune the viscosity and flow properties of the formulation to meet specific needs, ranging from sprayable disinfectants to semi-solid wound dressings or gel-based mucosal treatments [15,24,38]. These design strategies not only improve the physical and microbiological performance of the formulations but also ensure safe, efficient, and user-friendly application across clinical, pharmaceutical, and environmental settings [4,21].

## 2. Results and Discussion

### 2.1. Standard Low-Oil Nanoemulsion: Droplet Size and Preliminary Antimicrobial Screening

**Figure 1:**
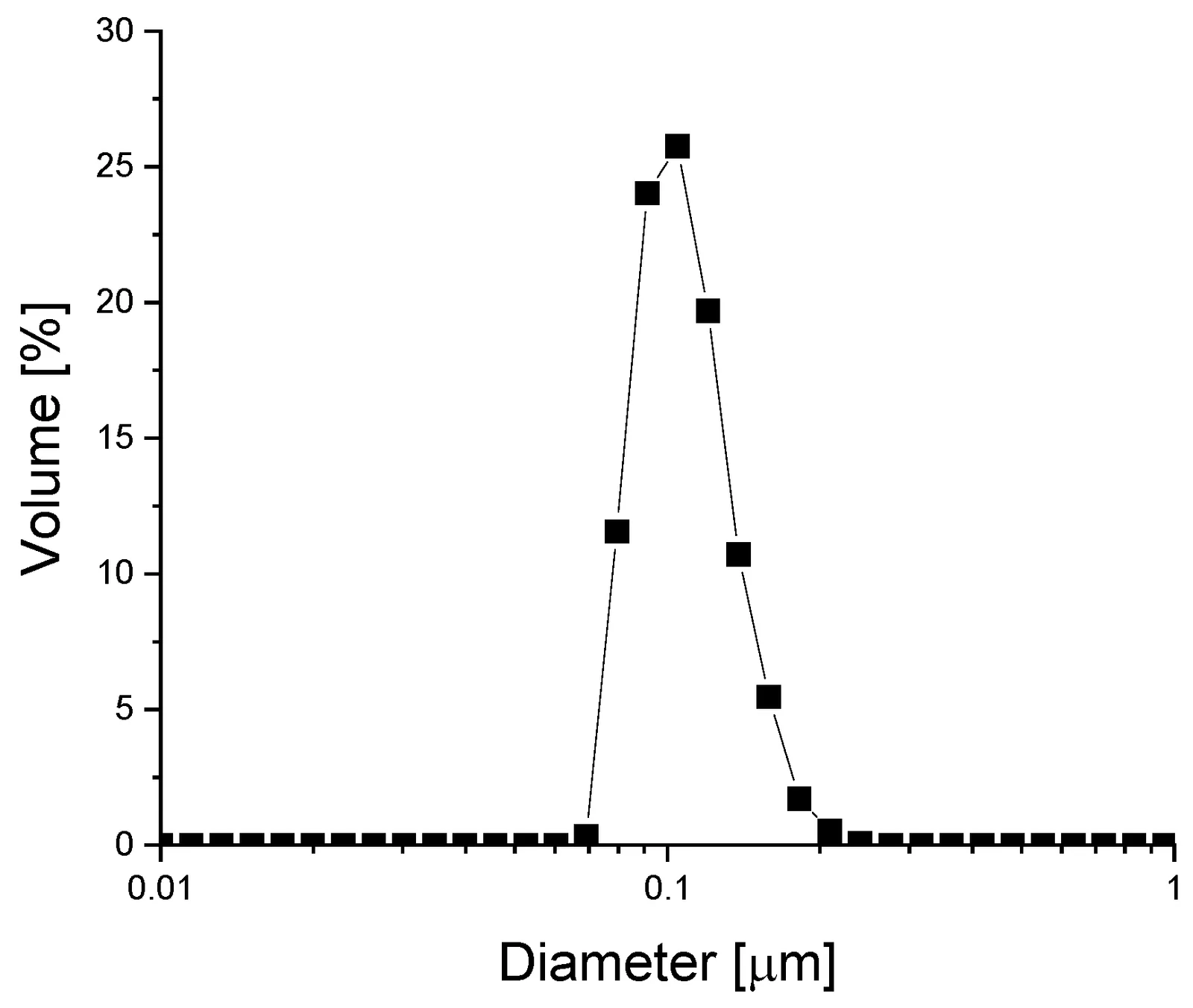
Droplet size distribution of the nanoemulsion formulated with 1.25 wt.% lavender oil.

**Figure 2:**
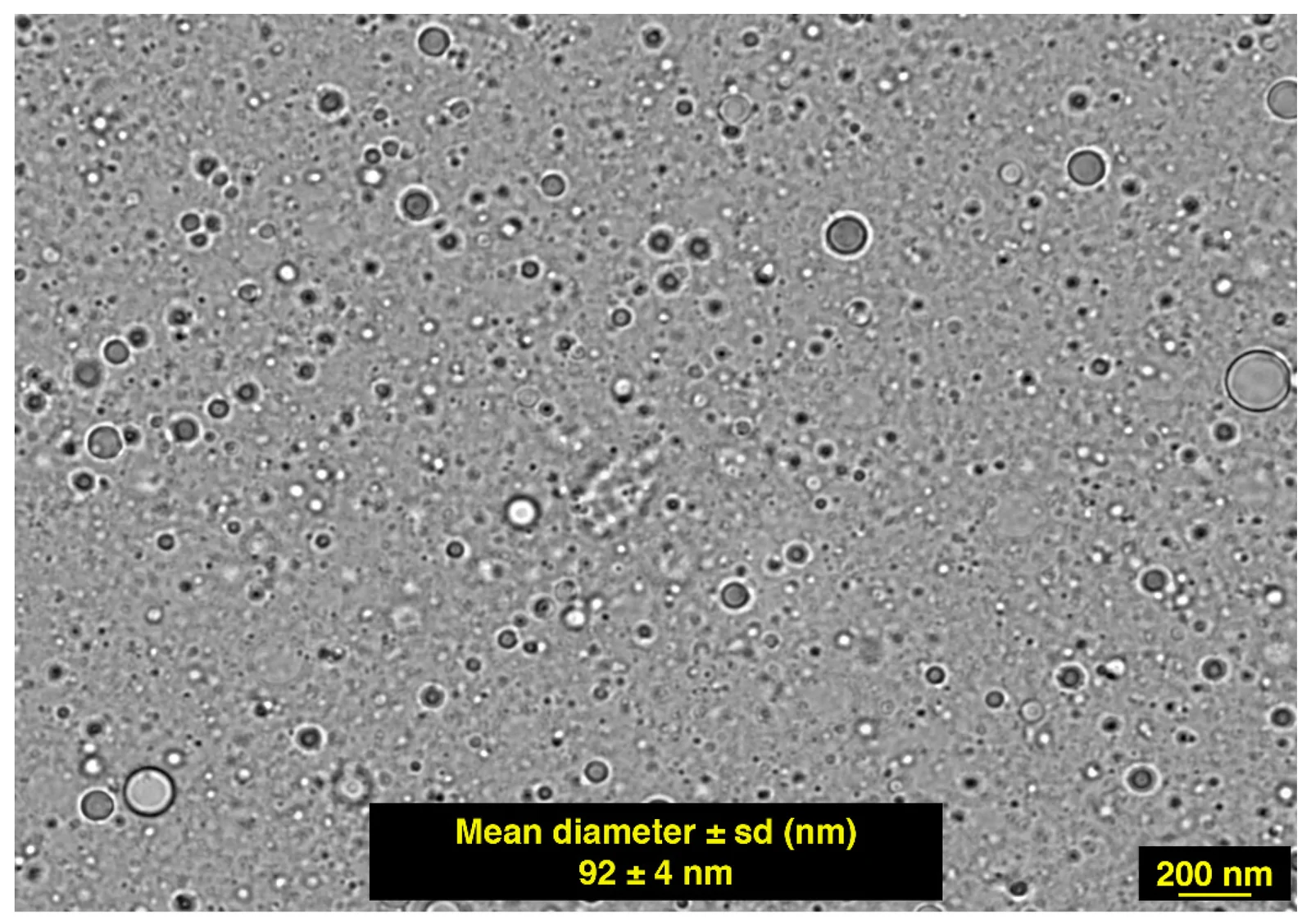
Optical microscopy image of lipid-based nanoemulsion containing lavender essential oil, prepared by high-shear mixing and ultrasonication (1:3 SOR; 70% amplitude; 15 min).

The nanoemulsion was prepared using a high-energy method combining high shear mixing and ultrasonication. The resulting formulation was analyzed by laser diffraction and optical microscopy to evaluate the average droplet size (see Figure 1 and Figure 2). Figure 1 presents a droplet size distribution that is confined to the nanometric range, characterized by a Sauter mean diameter (D_3,2_) of 114 nm and a volume-weighted mean diameter (D_4,3_) of 121 nm. The slight variation between these diameters suggests a minimal presence of larger droplets, indicating a uniform droplet population. This conclusion is reinforced by a span value of 0.563, which reflects a narrow size distribution. These findings demonstrate the success of the combined high-shear mixing and ultrasonication process in creating a finely dispersed nanoemulsion with negligible coarse droplets.

Results from laser diffraction were consistent with microscopy observations, indicating a mean droplet diameter of 92 ± 4 nm. Although the microscopic diameter was slightly lower than the Sauter and volume-weighted diameters obtained via laser diffraction, all measurements confirmed that the formulation predominantly contained droplets within the nanometric range. This discrepancy can be attributed to the differing principles of the techniques. Laser diffraction provides an ensemble measurement where larger droplets exert a proportionally greater influence, particularly on D_4,3_, whereas image analysis evaluates a limited number of droplets within selected two-dimensional microscopic fields. Consequently, microscopy may underestimate the contribution of the largest droplets. Nonetheless, the relatively close values obtained using both methods support the formation of a homogeneous and finely dispersed nanoemulsion. Microscopy qualitatively confirmed the finely dispersed and relatively homogeneous appearance of the nanoemulsion. However, given that the droplet dimensions were close to or below the resolution limit of conventional optical microscopy, laser diffraction results should be considered the primary quantitative measurement of droplet size.

A preliminary antimicrobial screening was conducted exclusively on the standard low-oil nanoemulsion. This formulation exhibited minimum inhibitory concentration (MIC) and minimum bactericidal concentration (MBC) values of 0.375 mg/mL against *E. coli*. Notably, an individual assessment of the formulation components revealed that lavender essential oil, when tested independently, did not demonstrate antimicrobial activity within the tested concentration range. In contrast, benzalkonium chloride exhibited both inhibitory and bactericidal activity at lower concentrations. Consequently, the antimicrobial effect observed in the standard nanoemulsion is primarily attributed to the presence of benzalkonium chloride, although the potential contribution of the entire formulation cannot be entirely dismissed. Conversely, the standard nanoemulsion did not inhibit the growth of *Serratia marcescens* under the tested conditions, whereas pure benzalkonium chloride demonstrated antimicrobial activity against this microorganism. As antimicrobial assays were not conducted on the Ghassoul-containing or high-oil structured formulations, these findings should be regarded solely as a preliminary characterization of the standard nanoemulsion and should not be used to infer conclusions about the gel-like systems.

### 2.2. Ghassoul-Clay-Induced Colloidal Gelation of Lavender Oil Nanoemulsions

**Figure 3:**
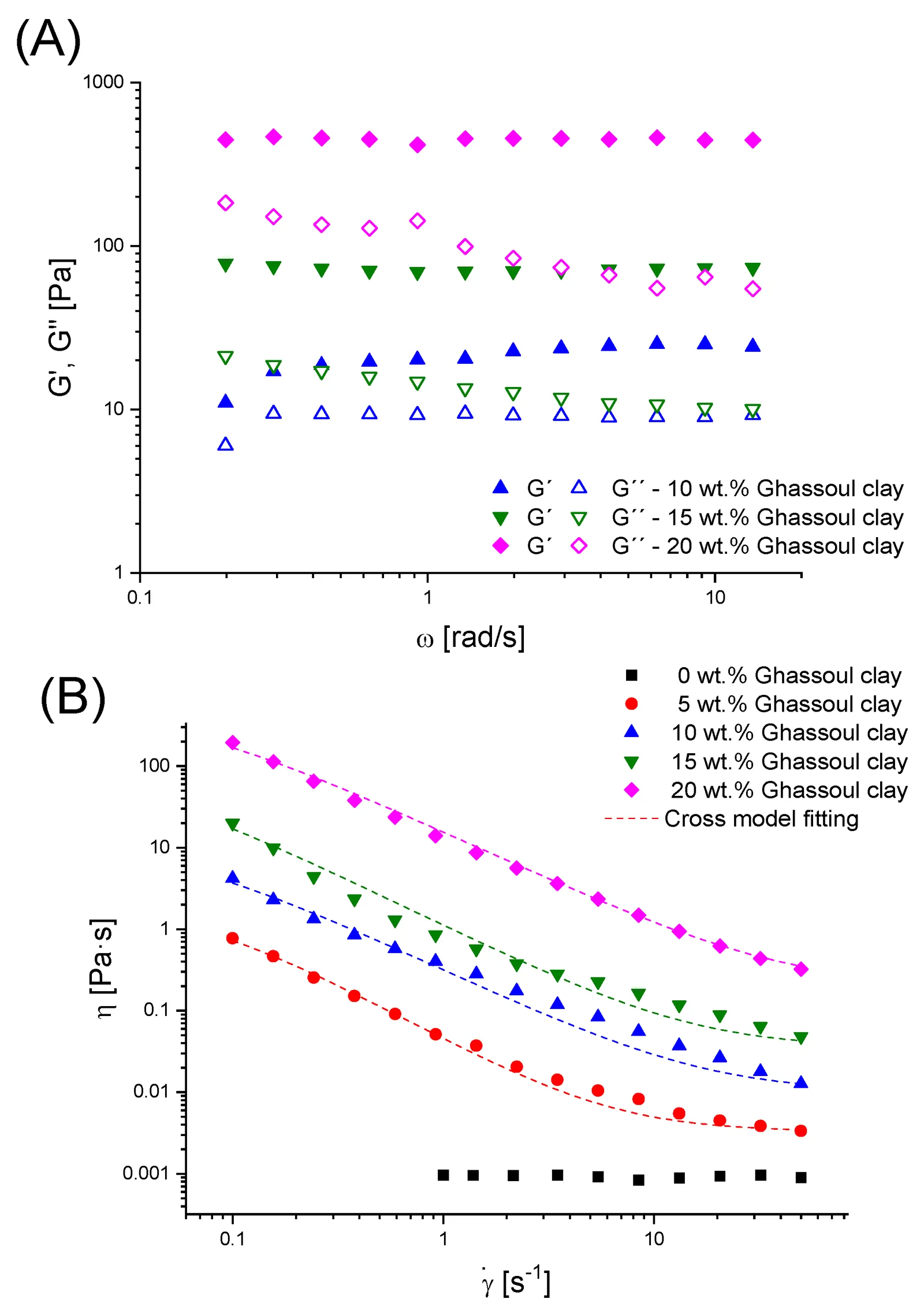
Mechanical spectra (**A**) and flow curves (**B**) of nanoemulsions containing Ghassoul clay. The dotted line represents the fit of the flow curves to the Cross model.

Ghassoul clay was incorporated into the nanoemulsion selected as a rheological modifier of the aqueous continuous phase, in different clay concentrations, 5 wt.%, 10 wt.%, 15 wt.% and 20 wt.%. The incorporation of Ghassoul clay did not significantly modify the droplet size of the standard nanoemulsion. The Sauter mean diameter (D_3,2_) remained within a narrow range, from 114 ± 8 nm (without clay) to 127 ± 12 nm (20 wt.% clay). Statistical analysis confirmed that these variations were not significant (*p* > 0.05), indicating that Ghassoul mainly structured the continuous phase without promoting measurable droplet coarsening. Rheological analysis was performed on the four samples obtained. Figure 3A and Figure 3B show, respectively, mechanical spectra and flow curves of these samples.

The mechanical spectra obtained from rheological analysis of nanoemulsions with different concentrations of Ghassoul clay (10 wt.%, 15 wt.%, and 20 wt.%) provided clear insight into the viscoelastic behaviour of the systems (Figure 3A). The nanoemulsions with 0 wt.% and 5 wt.% Ghassoul clay nanoemulsion was not able to be measured due to the limited linear viscoelastic range of the sample (critical stress below 0.5 Pa). All the samples observed exhibited typical viscoelastic characteristics, with both the storage modulus (G′) and the loss modulus (G″) increasing with frequency, indicating that the materials become more elastic under faster deformations. In addition, G′ was higher than G″ in all the frequency ranges studied, suggesting a gel-type structure. Increasing Ghassoul concentration resulted in a moderate increase in both G′ and G″, suggesting a slight reinforcement of the internal structure due to enhanced interactions among clay particles. At 20 wt.% clay content, there is a significant increase in G′, particularly at low frequencies, where it clearly dominates over G″ across the entire frequency range, highlighting a marked transition toward a gel-like, solid structure. The weak frequency dependence of G′ in this sample further confirms the formation of a strong, interconnected network within the nanoemulsion. The predominance of G′ over G″, together with the weak frequency dependence observed at high Ghassoul concentrations, indicates the formation of a weak colloidal gel rather than a merely viscous thickened nanoemulsion. This behavior suggests that Ghassoul platelets form a percolated particle network within the continuous phase, which acts as a stress-bearing structure and restricts both flow and sedimentation. These results demonstrate that Ghassoul clay plays a crucial role in modulating the mechanical properties of the system, with higher concentrations leading to more elastic, solid-like behavior, making this parameter essential for the design of nanoemulsions with tailored rheological performance for applications in cosmetic, pharmaceutical, or food formulations.

In Figure 3B, the flow curves represent the viscosity (η) as a function of shear rate ($\dot{\gamma }$) for nanoemulsions containing increasing concentrations of Ghassoul clay: 0 wt.%, 5 wt.%, 10 wt.%, 15 wt.%, and 20 wt.%. The clay-free nanoemulsion exhibited Newtonian flow behavior, with an approximately constant viscosity of 0.97 mPa·s throughout the shear-rate range studied, indicating the absence of a structured internal network. In contrast, the incorporation of Ghassoul clay caused a marked increase in viscosity and induced shear-thinning behavior, with the apparent viscosity decreasing as the shear rate increased. All samples containing the clay exhibit a pronounced shear-thinning behavior, where viscosity decreases with increasing shear rate, which is typical of structured and non-Newtonian fluids such as nanoemulsions containing dispersed solid particles. At low shear rates, viscosity is highest for the sample with 20 wt.% clay, followed by 15 wt.%, 10 wt.%, and 5 wt.%, indicating again that increasing clay content leads to a more interconnected microstructure that resists flow under low deformation. This fact remains consistent throughout the shear rate range, reflecting the reinforcing effect of Ghassoul particles on the rheological properties. The shear-thinning response becomes more prominent with higher clay concentrations, suggesting the breakdown of internal structures or flocculated networks under applied shear. These results confirm that clay content is a key factor in tuning the flow behavior of the system, offering control over viscosity and processability for potential applications requiring specific textural or handling characteristics.

The Cross model satisfactorily described the shear-thinning flow behavior of the Ghassoul-containing nanoemulsions. As shown in Table 1, the most pronounced concentration-dependent change was observed in the zero-shear viscosity (η_0_), which increased from 2 Pa·s at 5 wt.% Ghassoul clay to 12.2, 100, and 550 Pa·s at 10, 15, and 20 wt.%, respectively. Thus, increasing the clay concentration from 5 to 20 wt.% produced an approximately 275-fold increase in η_0_, evidencing the progressive formation of a highly interconnected structure capable of resisting deformation under quiescent or low-shear conditions. The characteristic Cross time constant, K, also increased from 15 to 35 s and reached an approximately constant value at clay concentrations of 15–20 wt.%. This increase suggests slower structural relaxation and a shift in the onset of shear thinning towards lower shear rates as the clay network became more developed. Simultaneously, the flow index, *n*, decreased from 0.41 to 0.18, indicating a progressively stronger deviation from Newtonian behavior and a more pronounced pseudoplastic response. In contrast, η∞ remained substantially lower than η_0_, showing that the clay-induced structure was largely disrupted under sufficiently intense shear.

**Figure 4:**
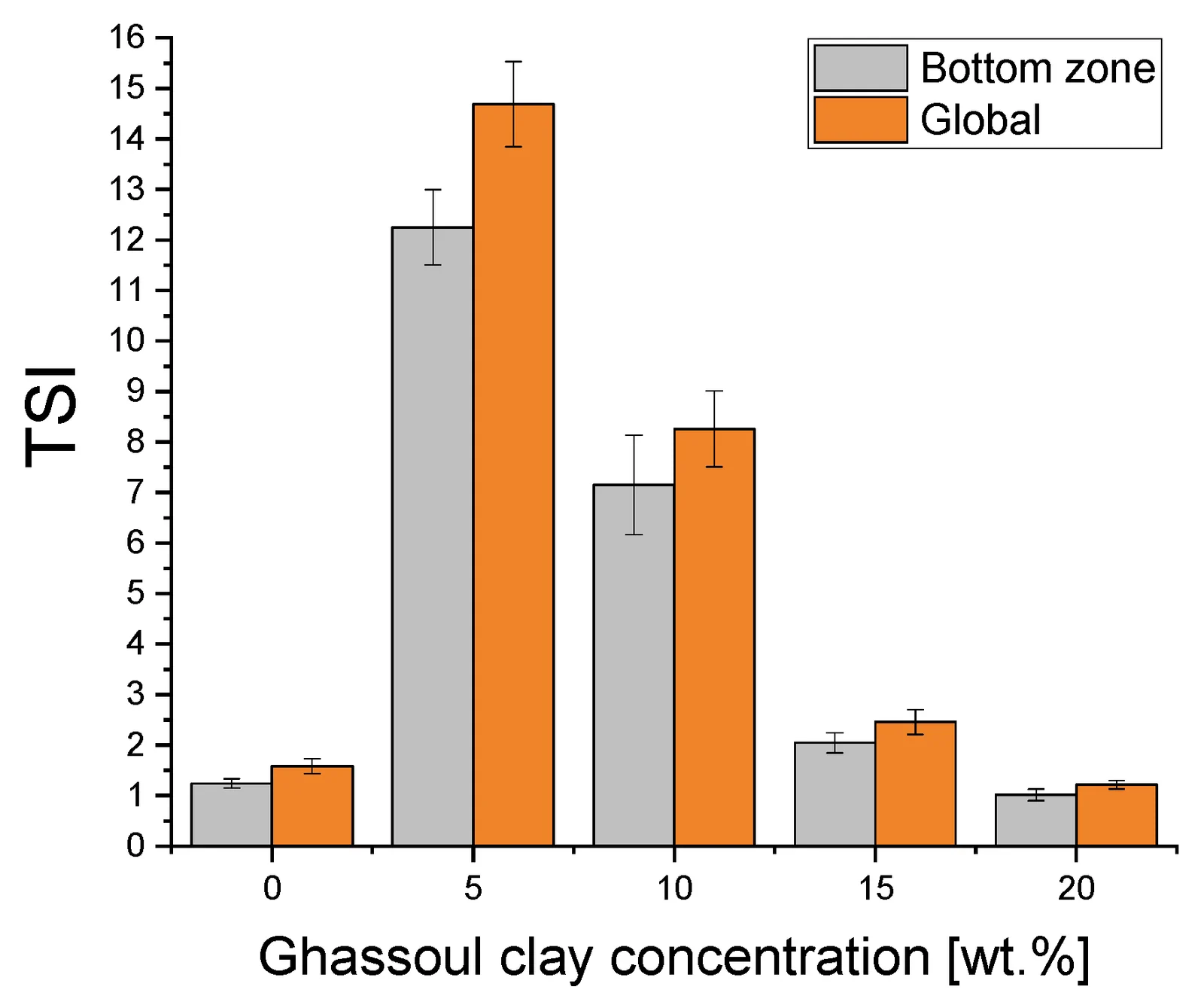
Turbiscan Stability Index (TSI) at 30 days values calculated for the bottom zone and the whole sample (global TSI) as a function of Ghassoul clay concentration. Values are expressed as mean ± standard deviation (*n* = 3). Statistical significance was evaluated by one-way ANOVA, with differences considered significant at *p* < 0.05.

Figure 4 shows the Turbiscan Stability Index (TSI) values calculated for the bottom region and for the whole sample as a function of Ghassoul clay concentration. The incorporation of 5 wt.% Ghassoul produced the highest global and bottom-zone TSI values, indicating pronounced physical changes during storage. A similar, although less intense, destabilization was observed for the formulation containing 10 wt.% clay. In both cases, the increase in TSI was mainly associated with the sedimentation of Ghassoul particles rather than with the migration or coalescence of the oil droplets. At these concentrations, the clay content was insufficient to form a continuous and mechanically resistant network capable of maintaining all mineral particles suspended within the aqueous phase. Consequently, part of the clay progressively accumulated at the bottom of the measurement cell, producing marked variations in the backscattering profile and increasing both the bottom-zone and global TSI values. This behavior may be interpreted through a Pickering-like contribution of Ghassoul clay. Although the direct adsorption of Ghassoul platelets at the oil–water interface was not measured in this study, clay particles may stabilize emulsions by partially associating with the droplet interface or forming a particle network within the continuous phase. In particle-stabilized emulsions, the concentration of the stabilizer is crucial, as it determines the amount of solid material available to cover newly formed oil–water interfaces and to construct a continuous stabilizing network. At low Ghassoul concentrations, the clay amount was likely insufficient to provide effective interfacial or structural coverage or to create a continuous network capable of immobilizing the dispersed particles. This insufficiency accounts for the higher TSI values observed at 5 and 10 wt.% Ghassoul, primarily due to clay sedimentation. Conversely, increasing the clay concentration to 15–20 wt.% enhanced particle availability, reinforced the continuous phase, and reduced particle migration, resulting in lower TSI values. Similar concentration-dependent effects have been documented for Pickering emulsions stabilized by biopolymer complexes, where higher stabilizer concentrations improved interfacial coverage, reduced droplet size, and enhanced physical stability [39]. In contrast, the formulations containing 15 and 20 wt.% Ghassoul displayed considerably lower TSI values. At these higher concentrations, the stronger three-dimensional network formed by the clay particles, together with the substantial increase in zero-shear viscosity, restricted particle settling and maintained a more homogeneous distribution throughout the sample. The 20 wt.% formulation showed the greatest resistance to sedimentation, with TSI values comparable to those of the clay-free nanoemulsion. These findings are consistent with the rheological results, which showed a pronounced increase in η_0_ and a more developed gel-like structure as the Ghassoul concentration increased. Therefore, a minimum clay concentration appears to be required to prevent Ghassoul sedimentation and obtain a physically stable, structured nanoemulsion. Thus, the improved stability observed at 15–20 wt.% Ghassoul can be directly related to the formation of a sufficiently strong clay network, whereas the weaker structures obtained at 5 and 10 wt.% were unable to prevent clay sedimentation. Analysis of the original backscattering profiles corroborated this interpretation. Significant variations were predominantly observed in the lower region of the measurement cell for formulations containing 5 and 10 wt.% Ghassoul, indicating particle migration and sedimentation within non-structured clay-rich domains. Conversely, formulations with 15 and 20 wt.% Ghassoul exhibited minimal backscattering changes along the sample height, suggesting that the reinforced clay network restricted particle displacement and enhanced physical stability. Transmitted light profiles did not yield additional relevant information due to the high turbidity of the samples, resulting in consistently low transmission throughout the measurements.

This behavior can be elucidated by considering the potential role of Ghassoul particles in the structuring of both the interfacial and continuous phases. In systems resembling Pickering emulsions, solid or composite particles may stabilize emulsions by adsorbing or associating at the oil–water interface, thereby enhancing interfacial rigidity and reducing droplet mobility. Furthermore, excess non-adsorbed particles may contribute to the formation of a three-dimensional network within the continuous phase, facilitating droplet bridging, particle–particle interactions, and the physical entrapment of the dispersed phase. In the current study, the observed progressive increase in G′ and zero-shear viscosity with increasing Ghassoul concentration indicates that the clay particles did not merely function as inert fillers but actively contributed to the formation of a stress-bearing structure. At low clay concentrations, this network was insufficiently developed to prevent sedimentation. However, at 15–20 wt.% Ghassoul, the reinforced continuous phase restricted particle and droplet mobility, thereby enhancing physical stability. Similar concentration-dependent effects have been documented in Pickering emulsion gels stabilized by biopolymer complexes, where particle-rich networks and interfacial coverage improved viscosity, water immobilization, and gel strength.

### 2.3. Oil-Loading-Induced Droplet Crowding and Gel-like Structuring

**Figure 5:**
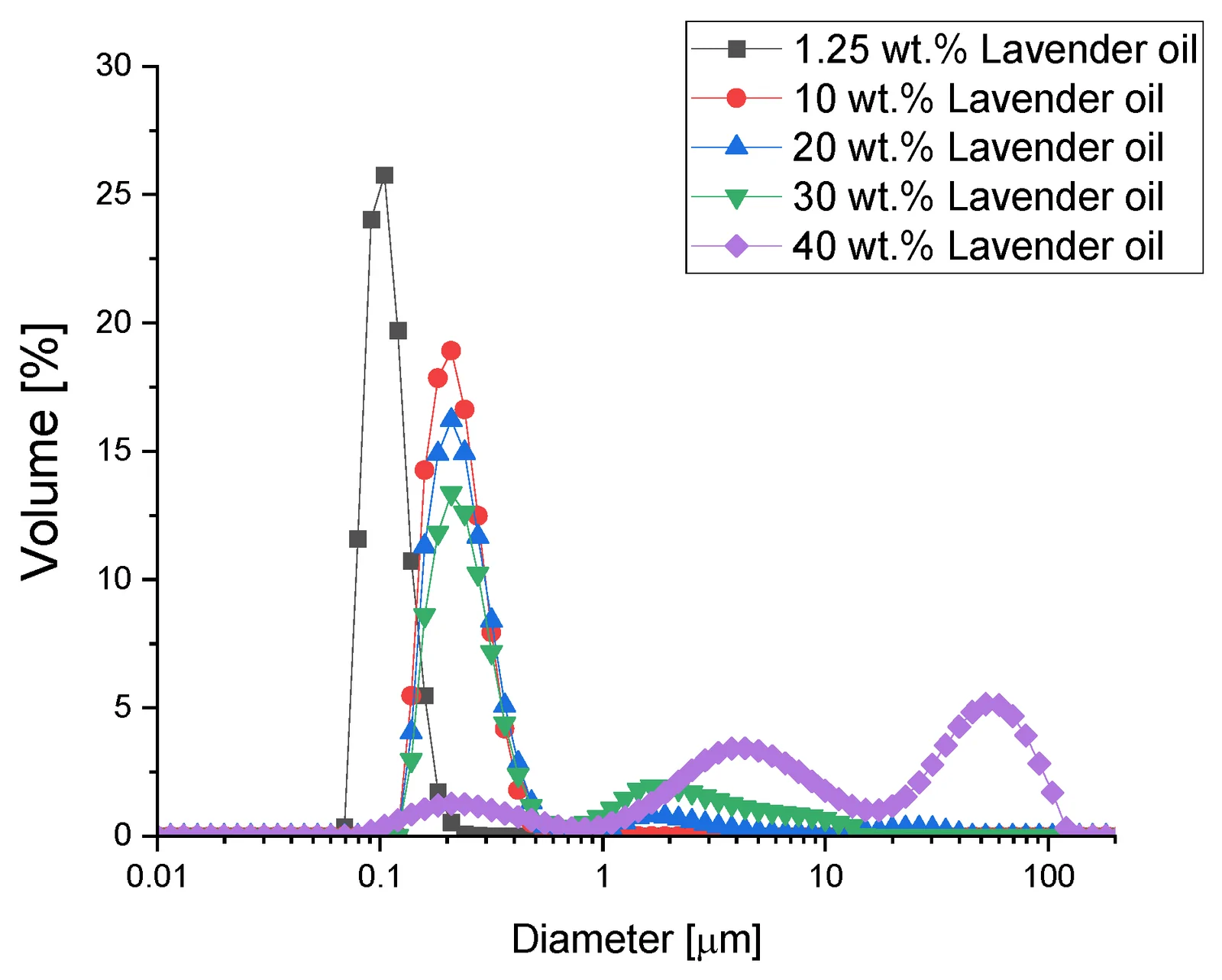
Droplet size distributions of emulsified systems prepared with different lavender essential oil concentrations.

Figure 5 shows that increasing the lavender essential oil concentration markedly affected both the mean droplet size and the width of the droplet size distribution. The standard nanoemulsion containing 1.25 wt.% oil exhibited a narrow and essentially unimodal distribution centred within the nanometric range. When the oil concentration was increased to 10 wt.%, the main peak shifted slightly towards larger diameters and became broader, although most of the dispersed phase remained within the submicron range. Further increases to 20 and 30 wt.% promoted a progressive broadening of the distribution and the appearance of secondary populations at micrometric sizes. This behavior became particularly evident at 40 wt.% oil, where a strongly multimodal distribution was observed, comprising a residual submicron population together with broad populations extending across the micrometric range. Therefore, the increase in oil loading progressively reduced the homogeneity of the dispersed system and favored the formation of larger droplets.

The quantitative parameters reported in Table 2 confirm these observations. The Sauter mean diameter, D_3,2_, increased progressively from 114 nm for the 1.25 wt.% formulation to 197, 267, 436, and 1343 nm for emulsified systems containing 10, 20, 30, and 40 wt.% lavender oil, respectively. The relatively moderate increase observed up to 30 wt.% indicates that a substantial fraction of the droplets remained within the submicron range. Nevertheless, the approximately twelve-fold increase in D_3,2_ between 1.25 and 40 wt.% oil demonstrates a clear loss of emulsification efficiency at the highest oil loading. Since D_3,2_ is sensitive to the surface area of the dispersed phase, its marked increase indicates a reduction in the specific interfacial area and the growing contribution of coarse droplets.

The span exhibited a similar trend, increasing from 0.563 at 1.25 wt.% oil to 1.351 at 10 wt.%, and then rising sharply to 11.007 and 14.765 at 20 and 30 wt.%, respectively. These elevated values suggest a significant broadening of the droplet size distribution, confirming the loss of homogeneity at high oil concentrations. In these highly polydisperse systems, the span should be regarded solely as a global descriptor of distribution width, rather than a comprehensive representation of the droplet population. As illustrated in Figure 5, the volume-based distributions indicate that the 20 and 30 wt.% formulations are not merely broad distributions but are multimodal systems comprising both submicron droplets and secondary micrometric populations. Consequently, the structural interpretation of these formulations was derived from the combined analysis of D_3,2_, span, and the complete droplet size distribution curves. Although the span decreased to 7.470 at 40 wt.% oil, this should not be construed as an enhancement in homogeneity. The complete distribution profile clearly demonstrates a highly multimodal and significantly shifted distribution, with a pronounced presence of coarse droplets. Therefore, the reduced span value at 40 wt.% likely indicates a redistribution of the dispersed volume among large droplet populations, rather than a return to a narrower or more uniform system. These results indicate that increasing lavender oil concentration above 10 wt.% progressively compromises the formation of a uniform nanoemulsion, and that the formulations containing 20–40 wt.% oil should be more appropriately described as highly polydisperse emulsified systems containing both submicron and micrometric droplet populations.

**Figure 6:**
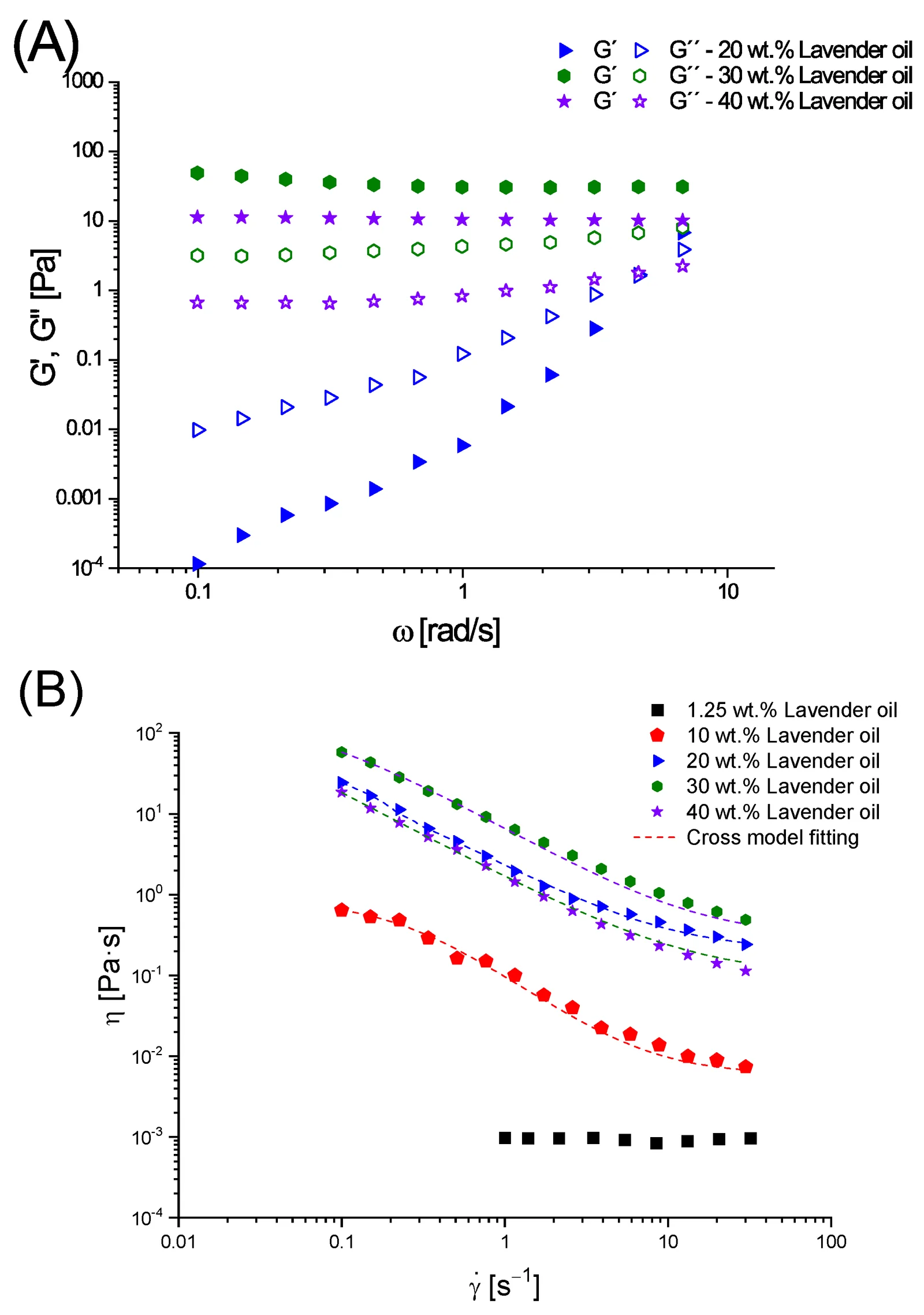
Mechanical spectra (**A**) and flow curves (**B**) of emulsified systems containing different lavender essential oil concentrations.

Figure 6A shows that lavender essential oil concentration strongly influenced the viscoelastic response of the emulsified systems. The formulation containing 20 wt.% oil exhibited predominantly viscous behavior, since the loss modulus (G″) remained higher than the storage modulus (G′) over most of the frequency range. Nevertheless, the progressive approach of both moduli at high frequencies suggests that elastic contributions became more relevant at shorter deformation times, although a fully developed gel-like network was not formed. Increasing the oil concentration to 30 wt.% produced a marked change in the mechanical response. In this formulation, G′ remained higher than G″ throughout the frequency range and both moduli showed relatively weak frequency dependence. These features are characteristic of a gel-like system with a continuous and mechanically resistant internal structure. Moreover, the 30 wt.% formulation exhibited the highest G′ and G″ values, indicating that this oil concentration promoted the strongest droplet-mediated network among the formulations studied. This reinforcement may be associated with increased droplet crowding, reduced inter-droplet distances and stronger hydrodynamic and colloidal interactions at high dispersed-phase volume fractions. The formulation containing 40 wt.% oil also displayed G′ values higher than G″ and a comparatively weak dependence on frequency, confirming the persistence of a predominantly elastic, gel-like response. However, both moduli were lower than those obtained for the 30 wt.% system. Therefore, the increase in oil concentration from 30 to 40 wt.% did not produce additional structural reinforcement. This non-monotonic behavior is consistent with the droplet size results, since the 40 wt.% formulation exhibited the largest Sauter diameter and a highly multimodal distribution. The formation of coarse droplets may reduce the number of droplets and the specific interfacial area available to establish an interconnected structure, while inefficient packing and possible recoalescence during processing may further weaken the network. The 30 wt.% oil formulation can therefore be interpreted as a droplet-mediated colloidal gel, in which the high dispersed-phase concentration reduces inter-droplet distances and promotes the formation of a continuous viscoelastic network. The lower moduli observed at 40 wt.% indicate that gel strength is not governed solely by oil concentration, but also by droplet size distribution and packing efficiency. Consequently, 30 wt.% oil appears to provide the most favorable balance between dispersed-phase concentration, droplet interactions and structural connectivity.

Figure 6B shows the apparent viscosity as a function of shear rate for emulsions containing different concentrations of lavender essential oil. The standard formulation containing 1.25 wt.% oil exhibited Newtonian behavior, with an approximately constant viscosity of 0.97 mPa·s, confirming the absence of an appreciable internal structure. In contrast, all formulations containing 10–40 wt.% oil displayed shear-thinning behavior, as evidenced by the progressive decrease in apparent viscosity with increasing shear rate. This response reflects the disruption and orientation of droplet aggregates or transient internal structures under flow.

The viscosity increased sharply when the oil concentration was raised from 10 to 20 wt.%, indicating a substantial enhancement of droplet crowding and inter-droplet interactions (see Table 3). The 30 wt.% formulation showed the highest viscosity over the entire shear-rate range, in agreement with its higher viscoelastic moduli and predominantly elastic behavior. This result confirms that the 30 wt.% system possessed the strongest and most interconnected microstructure. Conversely, the 40 wt.% formulation displayed lower viscosity than the 30 wt.% sample despite its higher oil content. This decrease may be related to its markedly larger and more heterogeneous droplet population, which can reduce packing efficiency and weaken the network formed by droplet–droplet interactions. Thus, the rheological response was not governed solely by the amount of dispersed phase, but also by the size distribution and organization of the droplets within the continuous phase.

The Cross model adequately described the shear-thinning behavior of the formulations containing 10–40 wt.% lavender essential oil, as shown by the close agreement between the experimental data and the fitted curves in Figure 6B. The most relevant change was observed in the zero-shear viscosity, η_0_, which increased from 0.8 Pa·s at 10 wt.% oil to 67.4 Pa·s at 20 wt.% and reached a maximum of 156 Pa·s at 30 wt.%. This pronounced increase confirms the progressive development of a resistant internal structure under quiescent and low-shear conditions. However, η_0_ decreased to 55.5 Pa·s at 40 wt.% oil, demonstrating that the highest oil loading did not generate the strongest structure. This non-monotonic trend agrees with the mechanical spectra and supports the conclusion that the 30 wt.% formulation possessed the most developed droplet network. The Cross time constant, K, increased from 4.3 s at 10 wt.% oil to approximately 15 s for the formulations containing 20–40 wt.% oil. Since the inverse of K is related to the characteristic shear rate at which structural breakdown begins, this increase indicates that the more concentrated systems started to exhibit shear thinning at lower shear rates and were therefore more sensitive to applied deformation. Simultaneously, the Cross exponent, *n*, decreased from 0.45 at 10 wt.% oil to values of approximately 0.13–0.15 at higher oil concentrations, evidencing a substantially stronger pseudoplastic response once the dispersed-phase concentration reached 20 wt.%. The infinite-shear viscosity, η∞, increased to 0.22 and 0.31 Pa·s for the 20 and 30 wt.% formulations, respectively, indicating that these systems retained comparatively high hydrodynamic resistance even after substantial structural disruption. In contrast, η∞ decreased to 0.01 Pa·s at 40 wt.% oil. This result may reflect a more complete breakdown of the heterogeneous structure under intense shear and the lower packing efficiency associated with its coarse and multimodal droplet population. Nevertheless, η∞ should be interpreted cautiously because a fully developed high-shear viscosity plateau was not clearly reached within the experimental shear-rate range.

The observed increase in storage modulus, apparent viscosity, and zero-shear viscosity can be attributed to the macroscopic effects of an increasingly interconnected network facilitated by droplets and particles. In structured Pickering emulsion gels, particles either adsorbed at the oil–water interface or situated within the continuous phase serve as physical junctions between droplets, promoting droplet bridging and the formation of a three-dimensional structure. This network limits droplet mobility during small-amplitude oscillatory deformation, thereby increasing G′, and also resists flow under low-shear conditions, leading to higher apparent viscosity and η_0_. This interpretation aligns with recent studies on Pickering emulsion gels. Xue et al. demonstrated that high-internal-phase Pickering emulsions stabilized by corn oligopeptide–chitosan complexes enhanced gel quality by facilitating pore filling, water immobilization, and the development of denser and more homogeneous microstructures [40]. Similarly, Ren et al. found that myosin-stabilized Pickering emulsion gels functioned as structural fillers, restricted water mobility, improved matrix homogeneity, and increased gel strength [41]. Although these systems were applied to surimi gel matrices, they support the broader concept that emulsion droplets and stabilizing particles can reinforce soft materials when homogeneously distributed and effectively integrated into a continuous network.

The non-monotonic rheological behavior observed at high lavender essential oil concentrations may be rationalized by the combined effects of dispersed-phase volume fraction, droplet size and polydispersity, packing efficiency, inter-droplet interactions, and interfacial properties. Increasing the oil concentration from 20 to 30 wt.% enhanced droplet crowding, reduced the average inter-droplet distance, and promoted the formation of a more interconnected stress-bearing structure, as evidenced by the increase in apparent and zero-shear viscosities and by the predominance of G′ over G″. Similar responses have been reported for highly concentrated emulsions, in which mechanical confinement, restricted droplet mobility, and transient droplet contacts can produce dynamically arrested and elastic structures [42,43]. Droplet size also contributes substantially to this behavior because, at a given dispersed-phase volume fraction, smaller droplets provide a larger total interfacial area and a greater number of droplets per unit volume, favoring more homogeneous packing and stronger collective interactions. Their higher Laplace pressure also makes smaller droplets less deformable, which may increase resistance to flow and reinforce the elastic response. Conversely, the increase in D_3,2_ from 436 nm at 30 wt.% oil to 1343 nm at 40 wt.%, together with the highly multimodal distribution observed at the highest oil loading, reduced the specific interfacial area and droplet number density and likely produced less efficient packing and a more heterogeneous network. In addition, the higher collision frequency and the lower efficiency of droplet disruption under these concentrated conditions may have favored recoalescence during processing, further contributing to the reduction in viscosity and viscoelastic moduli at 40 wt.% oil [44]. Interfacial film properties may also influence the macroscopic response, since viscoelastic interfaces can store mechanical energy and modify droplet deformation and droplet–droplet interactions [45]. Nevertheless, because interfacial rheology, droplet deformation, and coalescence were not directly measured, these mechanisms should be considered plausible interpretations rather than experimentally verified explanations. Classical models developed for less concentrated and more homogeneous emulsions, such as the Palierne model, may therefore have limited applicability to these highly concentrated and polydisperse systems [43,46]. Overall, the results indicate that 30 wt.% lavender oil provided the most favorable balance between dispersed-phase concentration, droplet size, packing, and structural connectivity, whereas the formation of larger and more heterogeneous droplets at 40 wt.% weakened the resulting network despite the higher oil content.

**Figure 7:**
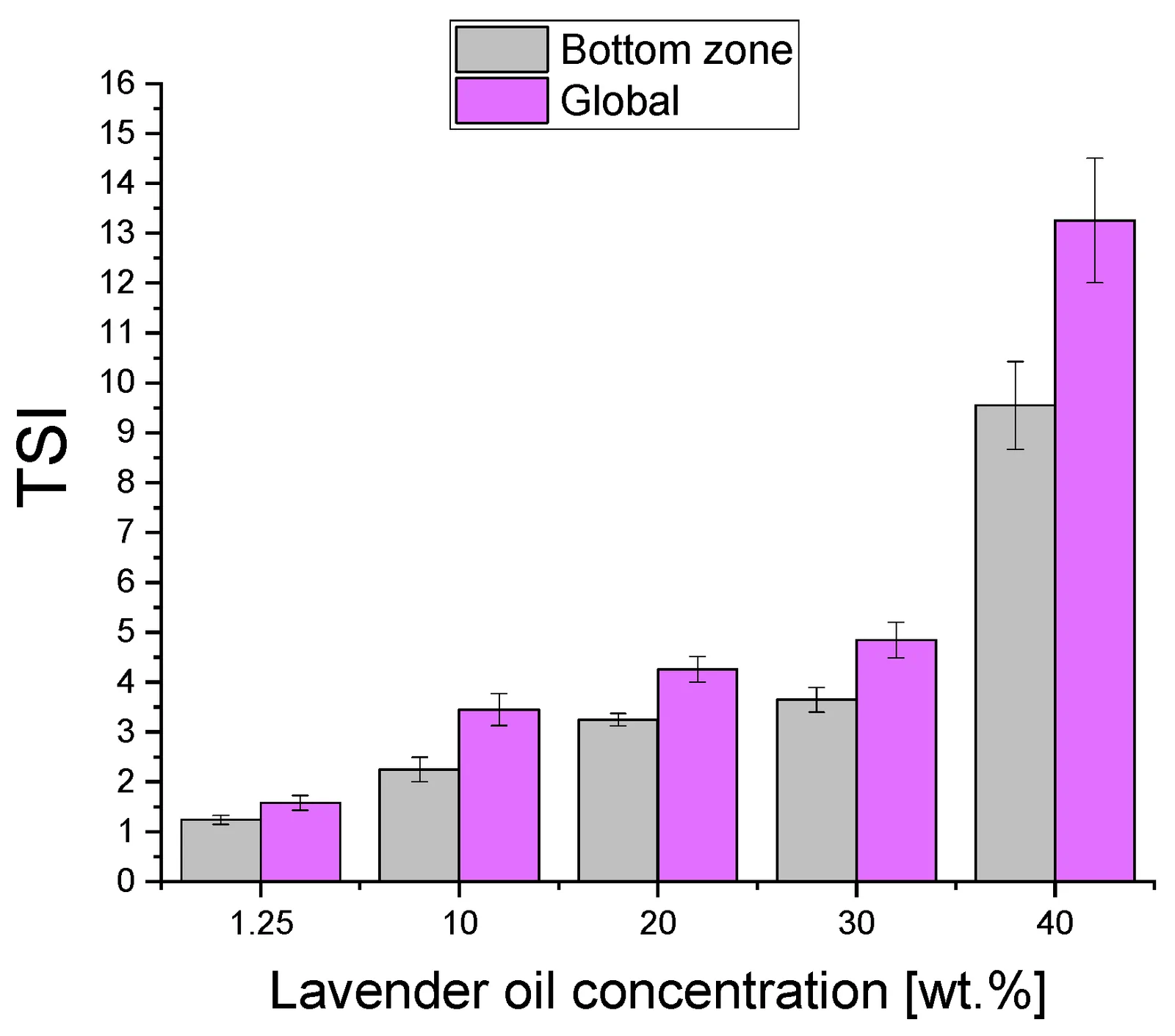
Turbiscan Stability Index (TSI) values obtained after 30 days for the bottom zone and the whole sample (global TSI) as a function of lavender essential oil concentration. Values are expressed as mean ± standard deviation (*n* = 3). Statistical significance was evaluated by one-way ANOVA, with differences considered significant at *p* < 0.05.

Figure 7 shows a progressive increase in both the global and bottom-zone Turbiscan Stability Index (TSI) as the lavender essential oil concentration increased, indicating a gradual reduction in physical stability. The formulation containing 1.25 wt.% oil exhibited the lowest TSI values and therefore the smallest changes in the backscattering profile, whereas the systems containing 10–30 wt.% oil showed intermediate destabilization. This trend became particularly pronounced at 40 wt.% oil, which displayed markedly higher global and bottom-zone TSI values. The increase in the bottom-zone TSI is consistent with the depletion of droplets from the lower part of the measurement cell due to upward creaming, while the higher global TSI indicates that structural changes occurred throughout the sample. These results agree with the droplet size data, since increasing oil concentration produced larger and more polydisperse droplets, particularly at 40 wt.%, thereby accelerating gravitational separation. Although the higher viscosity and gel-like behavior of the 30 wt.% formulation may partially restrict droplet migration, this stabilizing effect was insufficient at 40 wt.% because of its coarse and highly heterogeneous droplet population. The original backscattering profiles further confirmed the progressive destabilization of emulsified systems with increasing oil concentration. Formulations with low oil content demonstrated limited changes along the sample height, whereas high-oil systems, particularly the 40 wt.% oil formulation, exhibited more pronounced backscattering variations, consistent with droplet migration, creaming, and structural rearrangement. Similar to the Ghassoul-containing systems, transmitted light profiles were uninformative due to the high turbidity and opacity of the samples; thus, the discussion focused on backscattering-derived changes and TSI values.

**Figure 8:**
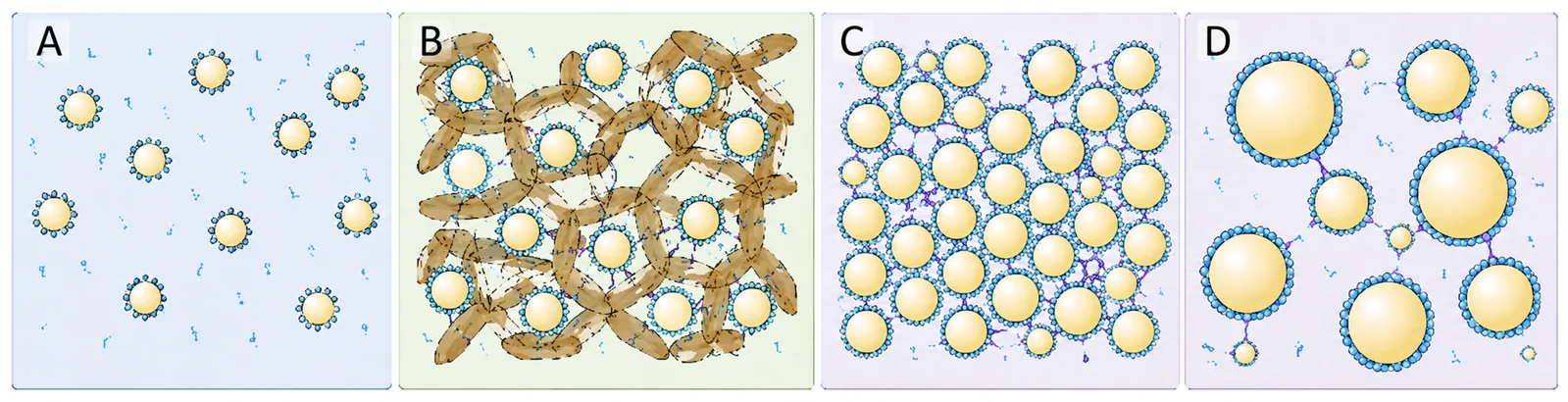
Schematic representation of the proposed structuring mechanisms in lavender essential oil emulsified systems: (**A**) low-oil nanoemulsion, (**B**) Ghassoul-clay-induced colloidal network, (**C**) droplet-crowding-induced gel-like structure at 30 wt.% oil, and (**D**) weakened structure at 40 wt.% oil due to droplet coarsening and polydispersity. The light-blue background represents the aqueous continuous phase, yellow spheres represent lavender essential oil droplets, blue interfacial layers represent the surfactant-stabilized oil–water interface, and brown plate-like elements represent Ghassoul clay particles or clay-particle networks. Colors are used only for schematic differentiation and do not indicate quantitative composition or scale.

The proposed structuring mechanisms are summarized in Figure 8. The standard formulation containing 1.25 wt.% lavender essential oil (A) behaved as a low-viscosity nanoemulsion, with dispersed droplets and no evidence of a continuous internal network. The incorporation of Ghassoul clay (B) promoted the formation of a particle-based colloidal network, which increased zero-shear viscosity, produced shear-thinning behavior and favored a gel-like viscoelastic response, especially at 15–20 wt.% clay. In contrast, increasing the lavender oil concentration induced structuring through droplet crowding. The 30 wt.% oil formulation (C) showed the strongest gel-like behavior, probably due to reduced inter-droplet distances and improved structural connectivity. However, at 40 wt.% oil (D), droplet coarsening and increased polydispersity weakened the network despite the higher dispersed-phase content. Therefore, the results indicate two different routes for converting lavender oil emulsified systems into gel-like materials: Ghassoul-induced particle networking and oil-loading-induced droplet crowding. From a structure–function perspective, the proposed mechanisms indicate that the functional properties of these systems were governed not only by composition, but also by the organization of droplets and clay particles within the continuous phase. Thus, droplet size, interfacial area, particle availability, and network connectivity determined the macroscopic response, including viscosity, storage modulus, and physical stability. This agrees with the general concept that the functionality of colloidal or bioactive systems depends strongly on their structural features and physicochemical interactions [47].

The proposed gelation and structuring mechanisms were primarily inferred from rheological and physical stability results, rather than direct macroscopic tests. Specifically, the predominance of G′ over G″, the weak frequency dependence of the viscoelastic moduli, the increase in zero-shear viscosity, and the reduced destabilization observed in certain formulations indicate the formation of gel-like or weak colloidal gel structures. However, the current study did not include photographs or vial-inversion tests, thus the shape-retention ability of these systems was not directly demonstrated. Future research should incorporate macroscopic visualization and inversion tests to provide direct evidence of self-supporting behavior.

In comparison to prior research on essential oil nanoemulsions, Pickering emulsions, and emulsion gels, this study primarily contributes by directly comparing two distinct structuring methodologies within a single lavender essential oil-based formulation platform. The Ghassoul-based approach enhances viscosity and induces gel-like behavior predominantly through clay-particle networking, without significantly altering droplet size. A comparable particle-networking strategy was recently reported for linseed oil Pickering emulsions containing ZnO and fumed silica/alumina, where interfacial ZnO shells and a continuous Aerosil network transformed a nearly Newtonian dispersion into a weak-gel system and reduced the Turbiscan Stability Index [48]. Conversely, the oil-loading method facilitates structuring via droplet crowding, while also leading to droplet coarsening and increased polydispersity at higher oil concentrations. Consequently, this research establishes new formulation criteria to differentiate between particle-network-driven and droplet-crowding-driven gel-like structuring in essential oil emulsified systems.

## 3. Conclusions

This study demonstrated that the concentration of Ghassoul clay and the loading of lavender essential oil significantly influence the structure, flow behavior, and physical stability of lavender oil-based emulsified systems. The standard formulation, containing 1.25 wt.% lavender oil, resulted in a finely dispersed nanoemulsion characterized by a narrow droplet size distribution and Newtonian behavior. The addition of Ghassoul clay did not notably alter droplet size but converted the low-viscosity nanoemulsion into shear-thinning and viscoelastic systems. An increase in clay concentration progressively strengthened the internal network, as evidenced by a significant rise in zero-shear viscosity and the predominance of G′ over G″ at higher concentrations. Formulations with 15 and 20 wt.% Ghassoul exhibited the highest resistance to clay sedimentation after 30 days, whereas 5 and 10 wt.% clay were inadequate for maintaining uniform suspension of mineral particles.

Increasing the lavender oil concentration led to a concurrent increase in droplet size, polydispersity, viscosity, and viscoelasticity, although the response was non-monotonic. The formulation with 30 wt.% oil displayed the strongest rheological structure, with the highest apparent and zero-shear viscosities and a distinctly gel-like response. In contrast, the 40 wt.% formulation exhibited larger, highly heterogeneous droplets and a weaker network despite its higher oil content. This behavior suggests that the rheological properties were governed not only by the dispersed-phase concentration but also by droplet size, packing efficiency, and structural connectivity. The TSI results further indicated that physical stability decreased with increasing oil concentration, with the 40 wt.% formulation showing the greatest destabilization after 30 days.

Ghassoul clay proved more effective than oil loading in increasing viscosity without altering droplet size, provided that a sufficiently high clay concentration was used to prevent sedimentation. Conversely, increasing oil concentration generated stronger structures only up to an optimal value of 30 wt.%, beyond which droplet enlargement and polydispersity diminished both network strength and stability. Therefore, only the standard 1.25 wt.% formulation and the Ghassoul-containing derivatives prepared from it should be considered nanoemulsions in the strict sense. By contrast, the high-oil formulations, particularly those containing 30 and 40 wt.% lavender oil, are better described as concentrated and highly polydisperse emulsified systems because of the presence of micrometric droplet populations. These findings offer valuable formulation criteria for designing lavender essential oil-based systems with adjustable flow, gel-like behavior, and application-dependent stability. Further studies, including interfacial rheology, zeta potential, and direct evaluation of droplet deformation and coalescence, would elucidate the mechanisms responsible for the observed structural changes. In addition, antimicrobial activity was evaluated only for the standard low-oil nanoemulsion and should therefore be considered a preliminary screening rather than a property of the structured formulations. Future work should assess the antimicrobial performance of the Ghassoul-containing and high-oil gel-like systems, since their increased viscosity, droplet organization, and physical structure may influence active release, diffusion, and contact with microorganisms.

From a gel-materials perspective, this work shows that essential oil-emulsified systems can be rationally converted into colloidal gels through two different structuring routes: clay-particle networking and droplet-crowding-induced gelation. The comparison between both approaches provides useful formulation criteria for designing gel-like essential oil delivery systems with tunable viscoelasticity, flow behavior, and storage stability. A further limitation is that the gel-like behavior proposed for the structured formulations was inferred from rheological and physical-stability data and was not supported by direct macroscopic evidence such as vial-inversion tests or shape-retention photographs. Future work should therefore include these macroscopic assessments to directly verify the extent of gel formation.

## 4. Materials and Methods

### 4.1. Materials

SoyPC from Lipoid GmbH (Ludwigshafen am Rhein-Maudach, Germany), *Lavandula angustifolia* essential oil from Bela Vizago Nature SL (Murcia, Spain), distilled water, benzalkonium chloride (BK) from Merck KGaA (Darmstadt, Germany), Ghassoul clay from Cosmundi (Saarburg, Germany). Test microorganisms used to examine the antimicrobial activity of nanoemulsion, and essential oil were *Escherichia coli* (ATCC 25922) and *Serratia marcescens* (ATCC14756).

### 4.2. LEO Nanoemulsion Preparation

Preparation of nanoemulsions by high energy method followed the reported procedure. Firstly, surfactants mixture of SoyPC and BK in 20:1 weight ratio was prepared in distilled water, at SoyPC concentration of 0.4 wt.%. Then, lavender essential oil in surfactant:oil weight ratio 1:3 (1.25 wt.%) was added in the aqueous phase using the Ultraturrax T-25 (IKA, Staufen, Germany) at 10,000 rpm for 60 s. Next, the pre-emulsion was introduced into the Ultrasonic Processor Model CV334 (Sonics & Materials, Newtown, CT, USA) and set with 70% amplitude and 15 min ultrasonication processing time, with a constant 5:5 pulse. While using the ultrasonic emulsification method, the emulsion has been introduced in a cold-water bath to prevent the emulsion from overheating.

Following the same procedure and employing the same ratios between components, emulsified systems with higher LEO concentrations of 10 wt.%, 20 wt.%, 30 wt.% and 40 wt.% were prepared. These high-oil formulations were not uniformly classified as nanoemulsions, since laser diffraction revealed the progressive appearance of broad and micrometric droplet populations at increasing oil loading.

### 4.3. LEO Nanoemulsion Characterization

The emulsified systems were characterized in terms of droplet size distribution, rheological behavior, and physical stability. In addition, the standard low-oil nanoemulsion was subjected to a preliminary antimicrobial screening.

#### 4.3.1. Droplet Size Distribution

Droplet size distributions were determined by laser diffraction using a Mastersizer 2000 particle size analyzer (Malvern Instruments Ltd., Worcestershire, UK). Before analysis, the emulsions were dispersed in distilled water until an appropriate obscuration level was reached, thereby minimizing multiple scattering effects. Measurements were performed at room temperature, and the results were expressed as volume-based droplet size distributions. The Sauter mean diameter, D_3,2_, and the volume-weighted mean diameter, D_4,3_, were determined. D_3,2_ is particularly sensitive to the specific interfacial area, whereas D_4,3_ is more strongly influenced by the presence of larger droplets. The width of the droplet size distribution was evaluated using the span parameter. In addition, the emulsions were observed under the microscope CX43 (Olympus Life Science, Tokyo, Japan). The images were taken from the microscope and have been analyzed using the program Image J (version 1.54s, National Institutes of Health, Bethesda, MD, USA). Considering each droplet size as circular particles, the diameter has been calculated and with all the values obtained, the average diameter (D) was also reached.

#### 4.3.2. Rheological Measurements

In this work, rheological measurements of nanoemulsions developed with different lavender essential oil or Ghassoul clay concentrations have been made with the Haake Mars 40 rheometer (Thermo Fisher Scientific, Waltham, MA, USA). Concretely, the rheology of the emulsified systems has been studied with 10 wt.%, 20 wt.%, 30 wt.% and 40 wt.% of *Lavandula a.* essential oil and at 5 wt.%, 10 wt.%, 15 wt.% and 20 wt.% of Ghassoul clay. For each sample, the dispersion was adequately treated with high shear mixer at 500–600 rpm for 30 min at room temperature, to achieve a homogeneous clay dispersion into nanoemulsion dispersant phase. The rheological study was divided into flow curves and frequency sweeps. The geometry used was a serrated plate-plate (60 mm diameter; 1 mm gap) to avoid the slip effect. The flow curve protocol was multi-step of 3 min/point. In addition, the protocol for frequency sweeps was from 20 to 0.05 rad/s at a stress in the linear viscoelastic range (LVR).

#### 4.3.3. Physical Stability

The physical stability of the emulsions was evaluated by multiple light scattering using a Turbiscan Lab Expert analyzer (Formulaction, L’Union, France). Freshly prepared samples were placed in cylindrical glass cells and analyzed without dilution. Physical stability was quantified using the Turbiscan Stability Index (TSI), which integrates the variations in transmission and backscattering profiles over time. Global TSI values were calculated over the entire height of the sample, whereas bottom-zone TSI values were obtained from the lower region of the measurement cell to assess destabilization phenomena occurring near the bottom. Lower TSI values indicate smaller changes in the optical profiles and, therefore, greater physical stability. The global and bottom-zone TSI values reported in Figure 4 and Figure 7 correspond to those obtained after 30 days of storage. TSI values were expressed as mean ± standard deviation from three independent measurements. Statistical analysis was performed by one-way analysis of variance (ANOVA) to evaluate the effect of formulation composition on bottom-zone and global TSI values. Differences were considered statistically significant at *p* < 0.05.

#### 4.3.4. Determination of the Minimum Inhibitory Concentration (MIC) and Minimum Bactericidal Concentration (MBC)

The MICs against the two microbial strains were determined following the standard microdilution protocol, according to CLSI method [38]. The essential oil and nanoemulsion were serially diluted in Mueller–Hinton broth (Condalab, Spain) using a two-fold dilution scheme. After inoculation, each well contained approximately 5 × 10^5^ CFU/mL. Microplates were incubated at 37 °C for 24 h. MICs were read as the lowest concentration of antimicrobial agent that completely inhibited the growth of the microorganisms as detected by the unaided eye. Since the nanoemulsion is formulated with benzalkonium chloride, the antimicrobial activity of this compound was also determined.

Bacterial suspensions containing nanoemulsions or benzalkonium chloride at concentrations equal to or greater than the minimum inhibitory concentration (MIC) were inoculated onto a solid medium. Minimum bactericidal concentration (MBC) was defined as the lowest concentration at which no bacterial growth was observed after subculture onto Mueller–Hinton agar plates.

## Figures and Tables

**Table 1:** Cross-model fitting parameters for the flow curves of the different nanoemulsions as a function of Ghassoul clay concentration.

Concentration [wt.%]	η_0_ [Pa·s]	η_∞_ [Pa·s]	K [s]	*n*
5	2 ± <0.1	0.2 ± 0.02	15 ± 1.3	0.41 ± 0.05
10	12.2 ± 0.7	0.01 ± 0.02	20 ± 1.9	0.22 ± 0.03
15	100 ± 2.9	0.03 ± 0.03	35 ± 3.1	0.20 ± 0.02
20	550 ± 12.6	0.01 ± 0.02	35 ± 3.3	0.18 ± 0.01

**Table 2:** Sauter mean diameter (D_3,2_) and span of the droplet size distributions of emulsified systems prepared with different lavender essential oil concentrations. Values are expressed as mean ± standard deviation (*n* = 3). Statistical significance was evaluated by one-way ANOVA, with differences considered significant at *p* < 0.05.

**Lavender Oil Concentration [wt.%]**	**D_3,2_ [nm]**	**Span**
1.25	114 ± 11	0.563 ± 0.045
10	197 ± 15	1.351 ± 0.105
20	267 ± 19	11.007 ± 0.785
30	436 ± 30	14.765 ± 1.163
40	1343 ± 112	7.470 ± 0.465

**Table 3:** Cross-model fitting parameters for the flow curves of emulsified systems prepared with different lavender essential oil concentrations.

Concentration [wt.%]	η_0_ [Pa·s]	η_∞_ [Pa·s]	K [s]	*n*
10	0.8 ± <0.1	0.006 ± 0.001	4.3 ± 0.8	0.45 ± 0.05
20	67.4 ± 3.7	0.22 ± 0.02	14.9 ± 1.4	0.13 ± 0.01
30	156 ± 5.2	0.31 ± 0.05	15 ± 1.2	0.14 ± 0.02
40	55.5 ± 5.4	0.01 ± 0.02	15 ± 1.3	0.15 ± 0.02

## Data Availability

The original data presented in the study are openly available in Zenodo at 10.5281/zenodo.21247303.
